# Truncated tau disrupts autophagy and endolysosomal systems

**DOI:** 10.1080/27694127.2024.2382659

**Published:** 2024-07-27

**Authors:** Saskia J. Pollack, Diane P. Hanger, Wendy Noble, Maria Jimenez-Sanchez

**Affiliations:** a Department of Basic and Clinical Neuroscience, Maurice Wohl Clinical Neuroscience Institute, Institute of Psychiatry, Psychology and Neuroscience, King’s College London, 5 Cutcombe Road, London, SE5 9RX, UK; b University of Exeter, Department of Clinical and Biomedical Sciences, Hatherly Laboratories, Prince of Wales Road, Exeter, EX4 4PS. UK

**Keywords:** Tau, tauopathies, Alzheimer’s disease, autophagy, dementia, endosomes, lysosomes, TFEB

Tauopathies are a group of neurodegenerative diseases characterised by cognitive and motor dysfunction, attributed to the accumulation of abnormal tau protein in the brain. Truncated forms of tau contribute to disease pathogenesis, including a C-terminal fragment of tau (Tau35) that we previously identified in the brain of individuals affected by tauopathy. Tau35 expression under the control of the human tau promoter in mice results in the deposition of highly phosphorylated aggregates of human Tau35 and endogenous mouse tau, progressive cognitive and motor deficits and impaired synaptic plasticity. However, the pathological mechanisms driven by tau fragments remain unclear. In our latest study, we linked disease-associated Tau35 with alterations in the autophagy and endolysosomal pathway, which have already been widely implicated in the pathogenesis of Alzheimer’s disease (AD) and other tauopathies.

Tau accumulation in tauopathy brain has been proposed to result from a failure of protein clearance mechanisms, including by autophagy. In addition, functional autophagy is compromised in a range of neurodegenerative disorders. However, to what extent tau fragments contribute to autophagy dysfunction is not fully understood. To address this, we investigated the effects of Tau35 on the accumulation of undegraded neutral lipid droplets, known substrates of autophagy, and expression of key autophagy and endolysosomal-related proteins [1]. We used cell lines stably expressing full-length human 2N4R tau (FL-tau) or Tau35, which lacks the amino terminal half of 2N4R tau, in Chinese hamster ovary (CHO), as well as primary neurons cultured from Tau35 transgenic mice. The CHO cell model allowed us to compare Tau35 with FL-tau in the absence of detectable endogenous tau, whereas in Tau35 neuronal cultures, the tau fragment is expressed together with endogenous tau.

We observed that Tau35 expression results in abnormal accumulation of neutral lipid droplets detected using BODIPY™ 493/503, both in Tau35-expressing CHO cells and in the soma of Tau35 neurons, suggesting a possible dysfunction in the capacity of autophagic degradation. Concomitantly, basal levels of the autophagosomal marker, LC3B (microtubule associated protein 1A/1B light chain 3B), were reduced, and the numbers of autophagosomes and autolysosomes decreased in Tau35-expressing CHO cells and Tau35 neurons, evidenced by quantification of mCherry-GFP-LC3B, a reporter that allows the measurement of the autophagic flux. Tau35-expressing CHO cells also exhibited significant reductions in the number of lysosomes, assessed by LAMP2 (lysosome-associated membrane protein 2) and cathepsin D immunofluorescence staining, as well as decreases in the number of LysoTracker-positive structures. Surprisingly, we identified discrepancies in the effects of Tau35 in CHO cells and primary neurons on lysosomal proteins; with Tau35 neurons showing an increase in LAMP2 and cathepsin D fluorescent labelling as well as increased LysoTracker-positive structures, with a notable defect in the motility of LysoTracker-positive organelles. We speculate that these discrepancies may be due to the differing amount of endogenous tau, supporting a model whereby Tau35 could potentially interfere with the physiological functions of other tau species. Despite discrepancies between the models, the expression of Tau35 results in alterations in autophagosomal and lysosomal proteins, which may lead to defective lysosomal clearance and would explain the accumulation of lipids observed in both model systems.

Tau35 expression also induced changes in early and late endocytic processes. Both Tau35-expressing CHO cells and Tau35 neurons harboured enlarged early endosomes, exhibited by increases in the size of EEA1 (early endosome antigen 1)-positive puncta, as well as increased fluorescent labelling of vesicles positive for RAB7, a marker for endosomes and lysosomes, but also autophagosomes. Endosomal abnormalities have been associated with AD, primarily in the context of amyloid precursor processing. Here, we provide evidence of a link between endolysosomal homeostasis dysfunction and tau pathology. Whether this is a direct effect of truncated tau on the endosomal system or is secondary to a defect in lysosomal function remains to be elucidated.

Given the alterations in lysosomal and autophagic organelles upon Tau35 expression, we investigated the possibility of a disruption in the function of TFEB (transcription factor EB), which controls the expression of genes regulating endolysosomal and autophagosomal biogenesis. We found that expression of either FL-tau or Tau35 repressed the ability of Torin 1 to stimulate mTOR mediated translocation of TFEB to the nucleus. This was demonstrated by a decrease in the nuclear-cytoplasmic ratio of TFEB in Tau35-expressing cell lines and by impaired translocation of TFEB-GFP to the nucleus in Tau35 neurons in response to Torin 1. FL-tau also diminished the ability of Torin 1 to induce TFEB nuclear translocation in CHO cells, suggesting a possible role for FL-tau as well as Tau35, in nucleocytoplasmic transport of TFEB. Over the years, TFEB activation has been explored as a potential therapeutic approach to enhance clearance of disease-associated cytotoxic proteins such as abnormal forms of tau. Our work emphasizes the importance of understanding the effect of tau on mechanisms controlling TFEB nucleocytoplasmic translocation and activation to further elucidate its contribution to neurodegenerative disease mechanisms.

In summary, our study shows that expression of disease-associated Tau35 results in defective autophagy and endolysosomal pathways (Figure 1). These findings provide insights into possible cellular and molecular mechanisms underlying tauopathies.

## Figures and Tables

**Figure 1:**
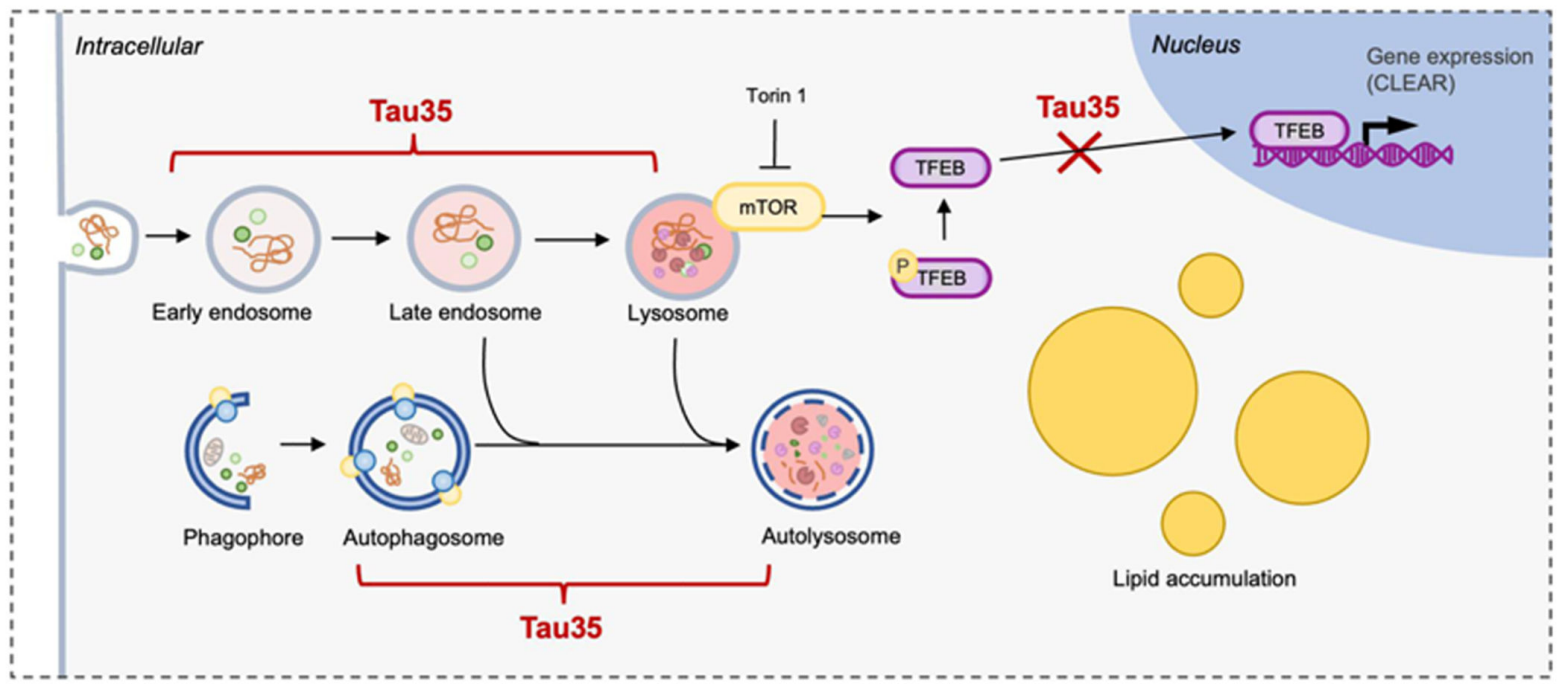
Illustration of potential sites of Tau35 interference with autophagy and endolysosomal pathways. Tau35 expression results in a reduction in numbers of autophagosomes and autolysosomes and accumulation of lipid droplets, concomitant to alterations in endolysosomal markers. Tau35 also impairs the Torin 1-induced nuclear translocation of TFEB.

## List of Abbreviations

AD: Alzheimer’s disease
CHO: Chinese hamster ovary
FL-tau: full-length human 2N4R tau
LC3: microtubule associated protein 1A/1B light chain 3B
TFEB: transcription factor EB
Tau35: 35 kDa fragment of 2N4R tau lacking the amino terminal
